# *PHKA2* mutation spectrum in Korean patients with glycogen storage disease type IX: prevalence of deletion mutations

**DOI:** 10.1186/s12881-016-0295-1

**Published:** 2016-04-21

**Authors:** Rihwa Choi, Hyung-Doo Park, Ben Kang, So Yoon Choi, Chang-Seok Ki, Soo-Youn Lee, Jong-Won Kim, Junghan Song, Yon Ho Choe

**Affiliations:** Department of Laboratory Medicine and Genetics, Samsung Medical Center, Sungkyunkwan University School of Medicine, 81 Irwon-ro, Gangnam-gu, Seoul, 135-710 Republic of Korea; Department of Pediatrics, Samsung Medical Center, Sungkyunkwan University School of Medicine, Seoul, Republic of Korea; Department of Laboratory Medicine, Seoul National University College of Medicine, Seoul National University Bundang Hospital, Seongnam, Republic of Korea

**Keywords:** Glycogen storage disease, Hepatomegaly, Inherited metabolic diseases, Korean, *PHKA2*

## Abstract

**Background:**

Molecular diagnosis of glycogen storage diseases (GSDs) is important to enable accurate diagnoses and make appropriate therapeutic plans. The aim of this study was to evaluate the *PHKA2* mutation spectrum in Korean patients with GSD type IX.

**Methods:**

Thirteen Korean patients were tested for *PHKA2* mutations using direct sequencing and a multiplex polymerase chain reaction method. A comprehensive review of the literature on previously reported *PHKA2* mutations in other ethnic populations was conducted for comparison.

**Results:**

Among 13 patients tested, six unrelated male patients with GSD IX aged 2 to 6 years at the first diagnostic work-up for hepatomegaly with elevated aspartate transaminase (AST) and alanine transaminase (ALT) were found to have *PHKA2* mutations. These patients had different *PHKA2* mutations: five were known mutations (c.537 + 5G > A, c.884G > A [p.Arg295His], c.3210_3212delGAG [p.Arg1072del], exon 8 deletion, and exons 27–33 deletion) and one was a novel mutation (exons 18–33 deletion). Notably, the most common type of mutation was gross deletion, in contrast to other ethnic populations in which the most common mutation type was sequence variant.

**Conclusions:**

This study expands our knowledge of the *PHKA2* mutation spectrum of GSD IX. Considering the *PHKA2* mutation spectrum in Korean patients with GSD IX, molecular diagnostic methods for deletions should be conducted in conjunction with direct sequence analysis to enable accurate molecular diagnosis of this disease in the Korean population.

## Background

Glycogen storage disease type IX (GSD IX) is caused by a general deficiency in phosphorylase kinase (PhK), which plays a major role in regulating the breakdown of glycogen. The PhK enzyme is composed of four copies each of four subunits (α, β, γ, and δ [also known as calmodulin]), which are encoded by *PHKA1* and *PHKA2* for the α subunits, *PHKB* for the β subunits, *PHKG1* and *PHKG2* for the γ subunits, and *CALM1*, *CALM2*, and *CALM3* for the δ subunits [1]. There are autosomal recessive forms of GSD IX (caused by mutations in *PHKB, PHKG1, PHKG2, CALM1, CALM2,* and *CALM3*), a X-linked liver form (caused by mutations in *PHKA2*), and a X-linked muscle form (caused by mutations in *PHKA1*). Specifically, the α subunits, which are encoded by the *PHKA2* gene in liver and by *PHKA1* in muscle, regulate the activity of the catalytic γ subunits, which carry out the function of PhK. Mutations in *PHKA2* are the most common cause of PhK deficiency [2].

The clinical manifestation of GSD IX stemming from *PHKA2* mutation is characterized by childhood onset of hepatomegaly, growth retardation, fasting ketosis, and fasting hypoglycemia [3]. Symptoms and biochemical abnormalities in GSD IX patients have been reported to improve with age [4]. However, several GSD IX patients possessing *PKHA2* mutations have been reported to progress to liver cirrhosis, or even present with cirrhosis at the time of diagnosis [4, 5].

GSD is a clinically and genetically heterogeneous group of diseases. Different types of GSD can sometimes be clinically indistinguishable; for example, both GSD VI and GSD IX often manifest with hepatomegaly with short stature [3]. Molecular diagnosis of GSD provides the advantage of avoiding invasive liver biopsy [6] and is an important starting point for appropriate therapeutic and monitoring plans [7]. Furthermore, molecular diagnosis allows determination of the inheritance pattern (autosomal recessive vs. X-linked) and DNA testing for other family members when the mutation in the proband is known.

The *PHKA2* gene is located at chromosomal locus Xp22.2-p22.1 and consists of 33 exons spanning more than 65 kb and encoding a protein of 1,235 amino acids [8]. Sequence analysis of this X-linked gene can detect small intragenic deletions/insertions and missense, nonsense, and splice site mutations in males and females, and lack of amplification by PCR prior to sequence analysis can suggest a putative exonic or whole-gene deletion on the X chromosome in affected males [9]. Understanding the mutation spectrum of causative genes in different ethnic groups could provide background knowledge to develop a tailored diagnostic approach for different patient populations [7].

To date, data on *PHKA2* mutations in Korean patients with GSD IX are available for only two cases. Thus, the aim of this study was to evaluate the mutation spectrum in Korean patients with GSD IX and compare it with previously reported mutation spectra in other ethnic populations.

## Methods

### Study population

From May 2010 to April 2015, blood samples from 13 unrelated Korean children were collected for *PHKA2* mutation analysis at Samsung Medical Center. The study population included two female patients with hepatomegaly and elevated aspartate transaminase (AST) and/or alanine transaminase (ALT) who were undergoing *PHKA2* sequencing because no pathogenic mutations in *G6PC* (for GSD Ia), which is known to a common causative gene of glycogen storage disease in the Korean population, were identified during diagnostic work up for their hepatomegaly [10, 11]. Blood samples from healthy individuals, who visited the health promotion center at Samsung Medical Center for regular health checkups without any clinical symptoms or signs of illnesses, and who volunteered for blood sampling, were also collected as negative controls for identified mutations. This study was conducted according to the guidelines laid down in the Declaration of Helsinki and all procedures involving human subjects were approved by the Institutional Review Board of Samsung Medical Center. Written informed consent was obtained from all subjects and/or their parents.

### PhK enzyme activity

PhK enzyme activity in erythrocytes was measured according to a previously described method [12, 13]. The results of enzyme activity were expressed as μmol/minute (min)/g hemoglobin (gHb). The reference range of phosphorylase b kinase enzyme activity in the laboratory was 100.0 to 250.0 μmol/hr/g Hb [12], which was validated using erythrocytes from healthy subjects. Each test was performed simultaneously in duplicate and with erythrocytes obtained from two different healthy subjects. Test results were accepted when the results of healthy control samples were within the reference range and the coefficient of variation of duplicated results was less than 10 %.

### Quantitation of liver glycogen

Liver glycogen was measured according to a previously described method [14]. Fresh liver samples, 45 μL in volume, were homogenized, heated at 95 °C for 5 min, and mixed with 45 μL of 0.1 M sodium acetate buffer (pH 4.8), and 9 μL of amyloglucosidase. Each homogenate was sonicated and incubated at 37 °C for 30 min and then at 95 °C for 5 min. Glycogen levels per gram of wet liver were calculated as glucose released by amyloglucosidase; i.e., glucose level after incubation with amyloglucosidase minus that without enzyme. The results were expressed as % glycogen/g wet liver weight. The reference range of liver glycogen in the laboratory was 1.0 to 6.0 % per gram of wet liver weight.

### *PHKA2* mutation analysis

Human genomic DNA was prepared from frozen white blood cells using a Wizard genomic DNA purification kit (Promega, Madison, WI, USA) according to the manufacturer’s recommendations. All 33 exons and the flanking regions of the *PHKA2* gene were amplified by polymerase chain reaction (PCR) using primers designed by the authors (Table 1) with a thermal cycler (Model 970; Applied Biosystems, Foster City, CA, USA). Direct sequencing of the DNA was performed using the ABI Prism 3100 Genetic Analyzer (Applied Biosystems) with the BigDye Terminator Cycle Sequencing-Ready Reaction Kit (Applied Biosystems). Nucleotides are numbered from the first adenine of the ATG translation initiation codon in the *PHKA2* cDNA Reference Sequence NM_000292.2.

**Table 1:** Primers used for PCR, sequencing, and multiplex PCR for *PHKA2* mutation analysis

Exon	Forward primer sequence	Reverse primer sequence	Product size (bp)
*PHKA2*			
1	CCATCCCAAGAACCGACTAA	GCAACAGTTAGGTCCCCTGA	395
2	AGGTCCCGGTCCTCATCTAC	GAGAGGCCTACACCCAAACA	367
3	AGCCACAGTGATCAGGAGGT	AATGACATGGAATGCCCACT	174
4	GCTGGGACATTTTAGGCAAG	CACATGGCCTGACACACTG	467
5	CCTTCCCTCTTTTCGGAGAT	GCAGTTTGTGTGTGGAGGTG	419
6	GGCTGCAGGAACATAAAGGA	CCAGGACGGAGCACTCTTAC	401
7	TTGCTTAATGAAAAAGGAACACC	CTAGCTTGTGAGGCCAGAGG	349
8	TGACTTCTCGCCTGAGGAAT	ACCTCATGGGGAACTGAGG	427
9	TATCTGCCTTGGTGGCTTTT	CCAGCTCACCGTCCCTACTA	434
10	TCAGTCAAGCATGGGAAACC	CTCTGCCCAAATTGCAGAAT	414
11	CCGATCGTGTTTAGCTCCTC	TCCCAAAGTGCTGGGATTAC	475
12	ATTGGCCTGGAGGATGAGTA	TGGACACACAAGGCTGAGAG	399
13	TGAATATGTTGAGCCCCAAA	CCCAGTTGCAATCAAGGTT	428
14	ATGTCACCAGGCAGAAGAGG	CCGCCTGCTTTAGTTTTTGT	353
15	GAAGAACCAAGCCCCAAAAT	ACGCCTGTCTCAAAAAGCAT	421
16	ACTGGGTGGATTGAAACGAG	AGAAGCCCCTTCAGTGCTTA	389
17	CGGGAATCTTCTATGCCAGA	TGGTTCACCTCCCTATGTCC	444
18	CCACATGGTTGTGCAAAAGT	CGGTTTTTAAACGGGCATT	441
19	GCTTGCTACCCATGGTCACT	GGGGCATTTTGTTGTCTTCT	364
20	GAGGCAAAGGTTGCAGTGAG	TGCAAGTCAGATTCCAGACAA	412
21	GAAAACTGGAGCACAGCACA	CCATGTCAGGATGCAATGAG	434
22	ACCACGTCCTGATGTTAGGC	ATGGGGCTCCTTCACAAGTA	449
23	TCCCTGTCTGGGTTGCTTAG	AGACGCATCCATGTGACAGA	382
24	TCTGTCACATGGATGCGTCT	TCTCCTGAGGCAGACACACA	303
25	ACAGCCTTCCTCAGAGTGGA	GGATGCTGGGTTCGAGATAA	321
26	TTTCAGCCCCAAAGCAATAG	ACACTGCGAGCAAGTCTCAA	435
27	CAGAGAAGGCCCTCATTGTC	GGACAGGGGTGTGTTCAGAT	376
28	CCATGAGAAATGCACTCGAA	ATAGAGCCGCCCTCTACACA	337
29	CTCTGCTGCTGCTTTCTGTG	GACGGAGAACAAAGCTCAGG	365
30	GTGGTGTTCTGGCATTTGTG	ATCCTCAGGGCTGTGTGTTT	383
31	TGTTCCATCGAAAACACAGC	TGATGCCAATAAATGCTGGA	436
32	GCTACGGTCACCCTTGGTTA	TTTTTCCCCATCATCTGTGA	410
33	CTCAGAAGGCCAAGGCTCTA	CTGATGGGACATGCTTTCCT	415
*MECP2* ^a^			
4	CGCTCTGCCCTATCTCTGAC	TCCCCTCGGTGTTTGTACTT	1061

^a^Used as amplification control of *MECP2* gene exon 4 located in Xq28 for multiplex PCR

To detect single or multiple exon deletions, a multiplex PCR method was performed using primers designed by the authors (Table 1). All tests were performed concurrently on negative control samples from healthy individuals.

Additionally, a comprehensive review of the literature on previously reported *PHKA2* mutations in Asian populations was conducted. The Leiden Open Variation Database (LOVD, http://www.LOVD.nl/PHKA2) and Human Gene Mutation Database (HGMD, h https://portal.biobase-international.com/cgibin/portal/login.cgi were checked for previously reported sequence variants. Variants identified in this study were checked through public databases. Common and rare variants present in the *PHKA2* gene in Korean population could be obtained from the Korean Reference Genome Database (http://152.99.75.168/KRGDB/) and compared with other ethnic populations through the Exome Aggregation Consortium (ExAC), which aggregates over 60,000 human exomes. The 1000 Genomes Project database (http://browser.1000genomes.org), the National Heart, Lung, and Blood Institute (NHLBI) Exome Sequencing Project (ESP) database (http://evs.gs.washington.edu/EVS), and the NCBI database of Single Nucleotide Polymorphisms (dbSNPs) were also checked for previously reported sequence variants.

The pathogenicity of missense variants was evaluated by *in silico* analyses using Sorting Intolerant from Tolerant (SIFT) (http://sift.jcvi.org/), and Polymorphism Phenotyping v.2 (PolyPhen-2) (http://genetics.bwh.harvard.edu/pph2/) prediction programs. Human Splice Finder software (http://www.umd.be/HSF3/) was used to predict splicing signals [15].

## Results

During the study period, six patients were identified as having *PHKA2* mutations. All six patients were male. Clinical and biological information at the time of diagnosis are detailed in Tables 2 and 3. Median age at first diagnostic work-up was 3 years (range 2–4 years). All patients presented with hepatomegaly and elevated AST and/or ALT as the first clinical features. Short stature, defined as height below the 3rd percentile for age by Korean Children and Adolescents Growth Standard [16], was observed in 33.3 % (2/6 patients, case 3 and 4) of GSD IX patients. The height of case 2, who had a missense mutation of c.884G > A (p.Arg295His), was within the 25th to 50th percentile. Among these six patients, only two had been tested for PhK enzyme activity in their erythrocytes, and both showed decreased PhK activity. Liver biopsies had been performed for five patients. Among them, the results of four (patients 3, 4, 5, and 6) were compatible with glycogen storage disease: Periodic acid–Schiff (PAS) staining was positive and PAS diastase stain was negative. One patient who was referred from an outside hospital was reported to be PAS^+^ in liver biopsy, but limited information was available because the liver biopsy had been performed at the outside hospital. All patients initiated uncooked cornstarch therapy after diagnosis of GSD. None of the patients experienced symptomatic fasting hypoglycemia at the time of diagnosis or during the follow-up period. Glucose monitoring, in which blood glucose was measured upon waking in the morning in a fasting state, identified one episode of asymptomatic fasting hypoglycemia (<70 mg/dL) [17] in each of case 2 and case 6.

**Table 2:** Summary of the clinical features of six GSD IX patients with *PHKA2* mutation

Patient no.	Sex	Age (yr)^a^	Hepato-megaly	Epistaxis	Short stature^b^	Hypo-glycemia	Hyper-uricemia^c^	Hyper-lactic acidemia^d^	Hyper-lipidemia^e^	CK increase^f^	Persistent hepatomegaly	Follow-up period (mo)
1	M	2	Yes	No	No	No	No	Yes	Yes	No	Yes	13
2	M	6	Yes	No	No	Yes^g^	No	Yes	No	No	No	26
3	M	4	Yes	Yes	Yes	No	No	Yes	No	No	No	178
4^h^	M	2	Yes	Yes	Yes	No	No	Yes	Yes	No	Yes^i^	48
5	M	2	Yes	No	No	No	No	Yes	Yes	No	Yes	33
6	M	4	Yes	No	No	Yes^g^	No	Yes	No	Not done	Yes	4

^a^Age at first diagnostic workup

^b^Short stature was defined as height below the 3rd percentile for age by Korean Children and Adolescents Growth Standard

^c^Uric acid level > 7.2 mg/dL for at least one measurement during follow-up period

^d^Lactic acid level > 2.2 mmol/L for at least one measurement during follow-up period

^e^Total cholesterol > 240 mg/dL and/or triglyceride >200 mg/dL for at least one measurement during follow-up period

^f^Serum creatine kinase >204 IU/L for at least one measurement during follow-up period

^g^Glucose monitoring in which blood glucose was measured upon waking in the morning in a fasting state identified one episode of asymptomatic fasting hypoglycemia (<70 mg/dL) during the follow-up period. The patient did not experience symptomatic hypoglycemia

^h^X-linked inheritance was identified by maternal *PHKA2* mutation analysis

^i^Mild improvement from two fingerbreadths to one fingerbreadth during follow-up period

**Table 3:** Mutations found in six Korean patients with GSD type IX

Case no.	Sex	Age^a^ (yrs)	PhK in erythrocytes^b^ (μmol/min/gHb)	Liver glycogen^c^	Liver biopsy	Identified mutation	Mutation type	Ref.
1	M	2	Not done	Not done	Not done	c.537 + 5G > A	Splicing	[6]
2	M	6	Not done	Not done	PAS(+)^d^	c.884G > A (p.Arg295His)	Missense	[8]
3	M	4	Not done	8.8 % (initial), 22.5 % (f/u)	c/w GSD, PAS(+), D-PAS(−)	c.3210_3212delGAG (p.Arg1072del)	Small deletion	[18, 19]
4	M	2	6.57	Not done	c/w GSD	Exon 8 deletion	Gross deletion	[13]
5	M	2	Activity below detection level	25.1 %	c/w GSD, PAS(+), D-PAS(−)	Exons 18–33 deletion	Gross deletion	This study
6	M	4	Not done	Not done	c/w GSD, PAS(+), D-PAS(−)	Exons 27–33 deletion	Gross deletion	[6]

*c/w* compatible with, *D-PAS* Periodic acid–Schiff diastase stain, *f/u* follow-up, *PAS* Periodic acid–Schiff stain, *PhK* Phosphorylase b kinase, *Ref* references

^a^Age at first diagnostic workup due to clinical presentation (all patients presented with hepatomegaly with elevated aspartate aminotransferase and alanine aminotransferase)

^b^Reference range of the laboratory was 100.0–250.0 μmol/min/gHb

^c^Reference range of the laboratory was 1–6 %/g wet liver weight

^d^Only limited information available on results of liver biopsy performed at outside hospital

**Fig. 1:**
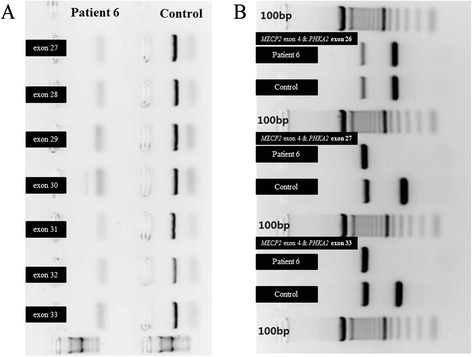
Example of a large exonic deletion identified in patient 6. **a** Lack of amplification of PCR products from exon 27 to exon 30 of the *PHKA2* gene in patient 6 compared with normal control. **b** Multiplex PCR results using control primers for amplification of *MECP2* gene exon 4 (1,061 bp) located in Xq28 and test primers for amplification of exon 26 (435 bp), exon 27 (376 bp), and exon 33 (415 bp)

Each of the six patients had a different *PHKA2* mutation. Among the six mutations, five were known mutations—c.537 + 5G > A [6], c.884G > A (p.Arg295His) [8], c.3210_3212delGAG (p.Arg1072del) [18, 19], exon 8 deletion [13], and exons 27–33 deletion (Fig. 1) [6]—and one was a novel mutation (exons 18–33 deletion). None of these mutations was observed in control population databases. The novel deletion mutation identified in case 5 was the largest deletion mutation (16 exons) ever reported among GSD IX patients, except for patients reported in the literature with full *PHKA2* deletion who had no paternal X-chromosome (LOVD). However, there was a lack of formal information on whether these patients had Turner syndrome, and therefore only one copy of *PHKA2*. Caution is required when using information from public databases and it is necessary to clarify the status of these patients.

**Fig. 2:**
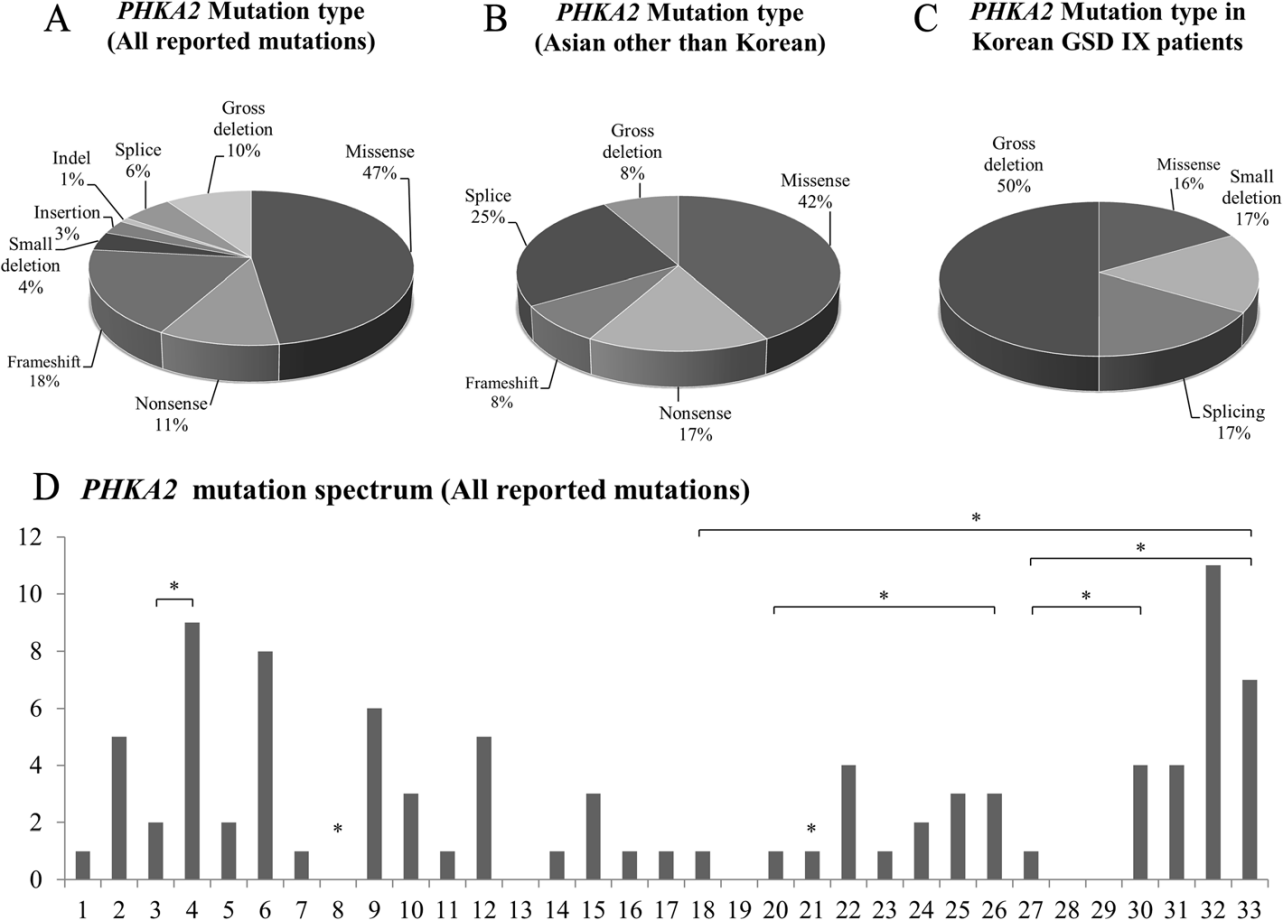
Summary of reported *PHKA2* mutation spectrum: **a** *PHKA2* mutation types among all reported mutations in all ethnic populations; **b** *PHKA2* mutation types identified in Asian GSD IX patients (other than Korean); **c** *PHKA2* mutation types identified in Korean GSD IX patients; **d** Number of reported *PHKA2* mutations in each exon (excluding gross deletions spanning more than one exon). The x-axis is the number of each coding exon and the y-axis is the number of identified mutations. *Gross deletion spanning more than one exon designated by additional asterisk and lines

Our review of the literature on *PHKA2* mutations in Asian populations with GSD IX is summarized in Table 4. Among 18 different *PHKA2* mutations reported in Asian populations, nine (50 %) were splicing or deletion mutations. The overall *PHKA2* mutation spectrum derived from a comprehensive literature review is summarized in Fig. 2. The most common *PHKA2* mutation type among other ethnic groups and Asian populations other than Koreans was sequence variants, such as missense, nonsense, or frameshift mutations.

**Table 4:** Reported *PHKA2* mutations in Asian patients with glycogen storage disease (GSD) type IX

Ethnicity	Exon number	Nucleotide change	Amino acid change	Mutation type	Reference
Point mutation					
Chinese	2	c.136delG	p.Asp46Ilefs*37	Frameshift	[31]
Japanese	4	c.346 T > G	p.Tyr116Asp	Missense	[32]
Japanese	6	c.578G > T	p.Gly193Val	Missense	[32]
Japanese	9	c.883C > T	p.Arg295Cys	Missense	[25]
Korean	9	c.884G > A^a^	p.Arg295His	Missense	[8], This study
Japanese	15	c.1489C > T	p.Arg497*	Nonsense	[25]
Japanese	16	c.1697A > T	p.Ile566Asn	Missense	[33]
Japanese	32	c.3505C > T	p.Gln1169*	Nonsense	[25]
Japanese, Chinese^b^	33	c.3614C > T	p.Pro1205Leu	Missense	[22, 24, 32, 34]
Splicing mutation					
Japanese	2	c.79-1G > T	Exon 2 skipping	Splicing	[25]
Korean	IVS5	c.537 + 5G > A	?^c^	Splicing	[6], This study
Japanese	25	c.2675A > G	Exon 25 skipping	Splicing	[32]
Chinese	30	c.3112-1G > A	Exon 30 skipping	Splicing	[31]
Deletion mutation					
Korean	30	c.3210_3212delGAG	p.Arg1072del	Small deletion	[18, 19], This study
Korean	8		Exon 8 deletion	Gross deletion	[13], This study
Korean	18–33		Exon 18–33 deletions	Gross deletion	This study
Japanese	20–26		Exon 20–26 deletions	Gross deletion	[29]
Korean	27–33		Exon 27–33 deletions	Gross deletion	[6], This study

^a^This mutation has been reported previously in two patients with GSD IX and is predicted to affect protein function by *in silico* analyses (SIFT and PolyPhen-2) and to affect splicing, potentially through activation of an exonic cryptic donor site, by both Human Splice Finder software and by a machine-learning technique that scores how strongly genetic variants affect RNA splicing [27]

^b^This mutation has been reported as a founder mutation in the Dutch population

^c^Although in vitro analysis for the splicing effect of c.537 + 5G > A was not performed, in vivo results confirming phosphorylase b kinase deficiency have been reported to constitute a pathogenic mutation in a patient with GSD IX in previous literature. This mutation was predicted to affect splicing, potentially through alteration of the wild-type donor site

## Discussion

**Fig. 3:**
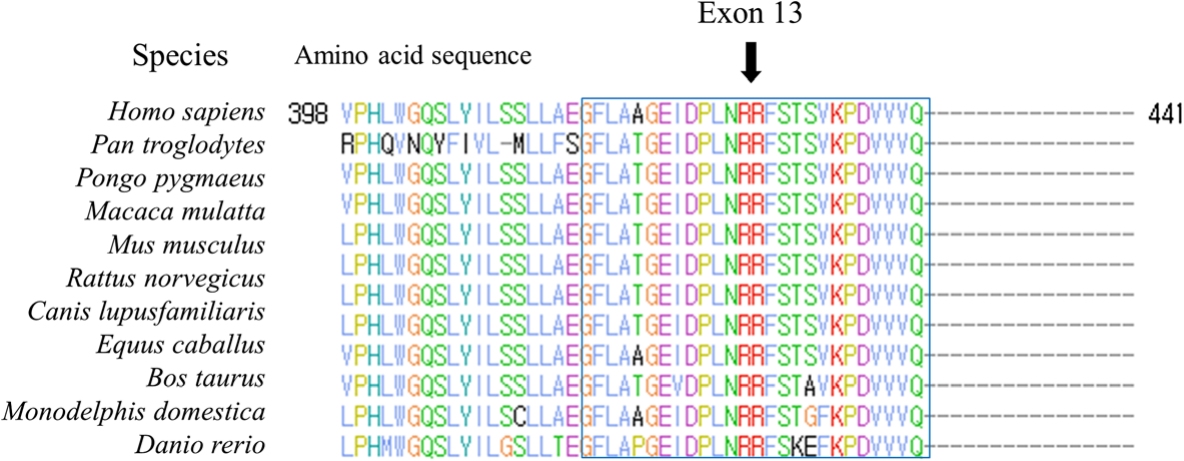
Evolutionary conservation of the amino acid residues for exon 13 on *PHKA2* gene. Except for several amino acid sites, such as amino acid 420, 423, and 432–434, other amino acid residues are strictly conserved in various mammals and zebrafish

In this study we examined the *PHKA2* mutation spectrum in Korean patients with GSD IX. GSD IX is associated with a deficiency in liver PhK (caused by mutations in *PHKA2, PHKB* and *PHKG2*) or the muscle form of PhK (caused by mutations in *PHKA1*). Mutations in *PHKA2* have been reported to be the most common cause of GSD IX (responsible for approximately 75 % of cases) [20, 21]. The second most common cause of liver PhK deficiency is mutations in *PHKG2* (autosomal recessive), followed by *PHKB* (also autosomal recessive) [3]. X-linked liver glycogenosis (XLG) caused by *PHKA2* mutations can be divided into two subtypes: XLG I with no detectable activity of phK in liver and peripheral blood cells, and XLG II with normal activity in peripheral blood cells and deficiency in the liver [8]. Although several female GSD IX patients with *PHKA2* mutations have been reported in other populations, including Western and other Asian populations [18, 22–24], only male patients were identified among our Korean patient population. The *PHKA2* mutation spectrum is known to be distributed across the entirety of *PHKA2* exons, with the exception of exon 13. Except for amino acids 420, 423, and 432–434, the protein sequence of PHKA2 is strictly conserved among various mammals and zebrafish (Fig. 3). Exon 13, which corresponds to amino acids 416 to 442, is in a six-hairpin glycosidase domain (amino acids 9 – 453) that contains pathogenic mutations including several missense mutations [2]. Although several missense variant alleles have been identified in exon 13 (ExAC), hemizygosity and lack of clinical information make interpretation of their clinical significance difficult. Further studies are needed to clarify the clinical significance of mutations in exon 13.

No recurrent mutations were observed among Korean GSD IX patients, although a missense *PHKA2* mutation of p.Pro1205Leu suggestive of a founder mutation has been reported among Dutch patients [22]. This mutation has also been reported in Japanese and Chinese patients, but has not been observed in the Korean population [22, 24, 25].

In this study, we identified one novel and five known *PHKA2* mutations. The missense mutation of c.884G > A (p.Arg295His) has been reported in a patient with unusually severe clinical manifestation with marked ketosis and hyperlipidemia [8, 26] In our study, patient 2 carrying the same mutation showed a relatively mild clinical manifestation, with asymptomatic hepatomegaly and asymptomatic hypoglycemia and height and weight within the 25th to 50th percentiles, although he had experienced hyperlactic acidemia. This mutation has been reported previously in a patient with XLG I who had a pronounced PhK deficiency in both liver and erythrocytes (PhK activity was 10.0 % of the control mean in both liver and erythrocytes) and in a patient with XLG II who had normal erythrocyte PhK activity (83.0 % of the control mean), therefore the significance of this mutation for biochemical function in specific tissues and clinical severity remains speculative [8]. This mutation was predicted to affect protein function by *in silico* analyses (SIFT and PolyPhen-2) and was predicted to alter splicing through activation of an exonic cryptic donor site by both Human Splice Finder software and by a machine-learning technique that scores how strongly genetic variants affect RNA splicing [27]. The c.537 + 5G > A mutation has been reported in a male GSD IX patient of European ancestry who presented with hepatomegaly and growth delay that presented from 9 months of age [6]. This mutation was predicted to affect splicing through alteration of the wild-type donor site. Although in vitro analysis of the splicing effect of c.537 + 5G > A was not performed, in vivo results confirmed phosphorylase b kinase deficiency in the patient’s lymphocytes (32 U/g, normal range 100–240 U/g) and erythrocytes (0.7 U/g Hb, normal range 4–10 U/g Hb) [6]. Clinical manifestations of the patient carrying this mutation were reported to include growth delay, mild fasting hypoglycemia, post-prandial lactatemia, and elevated serum triglycerides, with age at onset of 9 months [6]. In our study, patient 1 carrying the same mutation had post-prandial lactatemia and elevated serum triglycerides in common, but no fasting hypoglycemia or growth delay. A small deletion mutation of c.3210_3212delGAG (p.Arg1072del) has been reported in a Finnish male GSD IX patient with hepatomegaly without short stature or hyperlactic acidemia [18] whose age at onset was 16 months. That patient was diagnosed with GSD IX by molecular diagnosis, but a PhK enzyme activity test was not performed [18]. In this study, the patient with the same mutation was of short stature and his liver glycogen concentrations increased over time (8.8 %/g wet liver weight at initial diagnosis and 22.5 %/g wet liver weight at 2.5 year follow-up; reference range 1–6 %/g wet liver weight). His age of onset was 4 years and he experienced hyperlactic academia, which was different from the Finnish male. A gross deletion mutation of exons 27–33 has been reported in a male GSD IX patient of European ancestry with PhK deficiency diagnosed by markedly decreased phosphorylase activities in his lymphocytes and erythrocytes, whose age at onset was 8 months [6]. Because PhK (defective PhK activities cause GSD IX) activates glycogen phosphorylase b (defective phosphorylase activities cause GSD VI) by phosphorylation, patients with GSD IX can have decreased phosphorylase activities [6]. Molecular studies and analysis of PhK are needed to make the correct diagnosis and avoid misdiagnosis of GSD VI [28]. The male patient in the previous literature was reported to have post-prandial lactatemia with elevated serum triglycerides [6]. In this study, patient 6 carrying the same large deletion had asymptomatic fasting hypoglycemia and post-prandial lactatemia but no elevated serum triglycerides. His age at first diagnostic workup was 4 years.

Of note, most mutations in Korean GSD IX patients were deletion or splicing mutations, except for one known missense mutation of c.884G > A (p.Arg295His) [8]. Fifty percent of Korean GSD IX patients had gross deletion mutations, and 83.3 % had splicing or deletion mutations that were different from those reported in other ethnic populations. These results suggest that laboratory tests for large deletions in *PHKA2* should be included in the variety of methods that may be used such as quantitative PCR, long-range PCR, multiplex ligation-dependent probe amplification, and that chromosomal microarrays including this gene/chromosome segment should be used as the first approach for Korean patients suspected to have GSD IX [9]. In this study, the presence of deletions in male GSD IX patients was initially implied by the lack of amplification of exons by PCR and additional multiplex PCR was then was employed to better define the deletion. Although only male patients had *PHKA2* mutations in this study, multiplex PCR would be necessary to detect deletions in females with suspected GSD IX [9]. Methods to detect deletions would be important for patients of all ethnicities [6, 9, 29].

Historically, diagnosis of GSD was based on enzymatic defects of each type of GSD or on liver histopathology compatible with GSD [6]. Recent molecular diagnostic approaches based on mutation analysis for disease-causing genes associated with each type of GSD provide the advantages of avoiding invasive liver biopsy and allowing differentiation between several types of GSD with similar clinical findings [6].

Because of its variable clinical manifestations with mild symptoms, underestimation of GSD IX is possible. For example, short stature was observed in 33.3 % of patients in this study, which was comparable to previous studies in European and Argentinian populations [18] but different from a Canadian study that reported no patients with short stature [3]. Furthermore, the effect of *PHKA2* mutations on tissues other than liver should be clarified through future studies. A recent study reported that genetic diagnosis for X-linked mental retardation revealed several *PHKA2* variants [30]. Although the natural history of GSD IX in Canadian patients has been reported, there are no reliable data on long-term outcomes for Korean GSD IX patients [3]. The study on 11 Canadian GSD IX patient with *PHKA2* mutations with a follow-up period ranging from 1 to 16 years reported improved, normalized, or stable liver enzymes in all patients, and development of likely liver adenoma in one patient at the 5-year follow-up [3]. Detailed long-term natural history studies of Korean patients with GSD IX caused by *PHKA2* mutation will be helpful to understand whether these patients are at increased risk of developing additional complications such as liver cirrhosis or hepatocellular carcinoma, or other conditions such as reproductive or mental disorders, later in life.

PhK deficiency is very complex and many genes play a key role in the GSD IX phenotype; the spectrum includes autosomal recessive forms of GSD IX (caused by mutations in *PHKB, PHKG1, PHKG2, CALM1, CALM2,* and *CALM3*) as well as the X-linked liver form (caused by mutations in *PHKA2*) and X-linked muscle form (caused by mutations in *PHKA1*). Only *PHKA2* mutations were studied in this study. Future studies involving comprehensive mutational analyses of multiple causative genes in the Korean population are needed.

## Conclusions

In this study we summarized the *PHKA2* mutation spectrum in Korean GSD IX patients and found that the most common mutation type was gross deletion. The present study expands our knowledge of the mutational spectrum in Korean GSD IX patients, which differs from that in other ethnic populations. Considering the *PHKA2* mutation spectrum in Korean patients with GSD IX, molecular diagnostic methods for deletions should be combined with direct sequence analysis to provide accurate molecular diagnosis of this disease.
